# VKORC1 and VKORC1L1: Why do Vertebrates Have Two Vitamin K 2,3-Epoxide Reductases?

**DOI:** 10.3390/nu7085280

**Published:** 2015-07-30

**Authors:** Johannes Oldenburg, Matthias Watzka, Carville G. Bevans

**Affiliations:** 1 Institute of Experimental Haematology and Transfusion Medicine, University Clinic Bonn, Bonn 53105, Germany; E-Mail: matthias.watzka@ukb.uni-bonn.de; 2 Im Hermeshain 6, Frankfurt am Main 60388, Germany; E-Mail: bevans@jhu.edu

**Keywords:** evolution, subfunctionalization, paralog, vitamin K, VKOR, VKORC1, VKORC1L1

## Abstract

Among all cellular life on earth, with the exception of yeasts, fungi, and some prokaryotes, VKOR family homologs are ubiquitously encoded in nuclear genomes, suggesting ancient and important biological roles for these enzymes. Despite single gene and whole genome duplications on the largest evolutionary timescales, and the fact that most gene duplications eventually result in loss of one copy, it is surprising that all jawed vertebrates (gnathostomes) have retained two paralogous VKOR genes. Both VKOR paralogs function as entry points for nutritionally acquired and recycled K vitamers in the vitamin K cycle. Here we present phylogenetic evidence that the human paralogs likely arose earlier than gnathostomes, possibly in the ancestor of crown chordates. We ask why gnathostomes have maintained these paralogs throughout evolution and present a current summary of what we know. In particular, we look to published studies about tissue- and developmental stage-specific expression, enzymatic function, phylogeny, biological roles and associated pathways that together suggest subfunctionalization as a major influence in evolutionary fixation of both paralogs. Additionally, we investigate on what evolutionary timescale the paralogs arose and under what circumstances in order to gain insight into the biological *raison d’être* for both VKOR paralogs in gnathostomes.

## 1. Introduction

**Figure 1:**
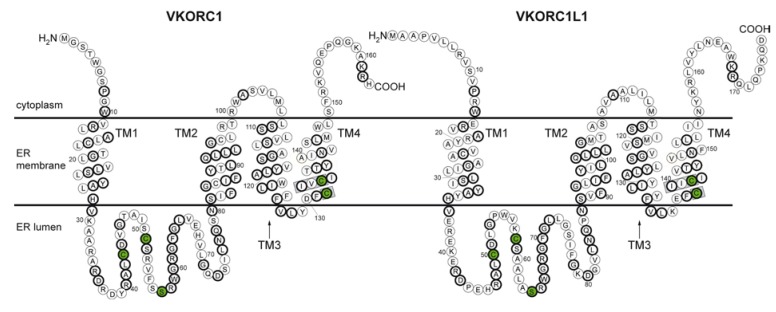
Primary protein sequence and predicted topology of human VKORC1 (left) and VKORC1L1 (right). Circles represent amino acid residues; bold circles indicate positions of sequence identity shared by both paralogs; green-filled circles, residues conserved among all VKOR family proteins; TM1–TM4, first through fourth transmembrane α-helices; gray-boxed regions, the catalytic CXXC active site motif.

Genomes of higher vertebrates possess two paralog genes, *VKORC1* and *VKORC1L1* (see Note 1 [1]), that encode enzymes unique in catalyzing de-epoxidation of vitamin K 2,3-epoxide (K>O), a product of post-translational modification of vitamin K-dependent (VKD) proteins [2,3]. VKD proteins are known to be essential for diverse physiological functions including hemostasis and coagulation [4,5]; bone development and homeostasis [6,7,8]; vascular homeostasis, remodeling and calcification [9,10,11,12,13]; cellular growth, survival, and signaling [14,15]; metabolic homeostasis [16,17]; and fertility [18]. While the respective VKORC1 and VKORC1L1 protein primary sequences share ~50% identity and highly homologous function (Figure 1), it is surprising that both genes have been maintained with high fidelity throughout over 400 million years of vertebrate evolution [3,19] (See also Bevans *et al.* [20] in this Special Issue). In the following review, we point out structural and functional similarities and differences between both paralog enzymes and explore phylogenetic relationships in order to construct a hypothesis that addresses the question “Why do vertebrates have two vitamin K 2,3-epoxide reductase (VKOR) enzymes?”.

### 1.1. Catalytic Function and Biological Roles of VKOR Family Enzymes

VKOR family homologs are expressed in the vast majority of the currently available sequenced genomes except those for all fungi and yeasts, and about half of the prokaryotic genomes available to-date, which almost always alternatively express DsbB oxidoreductases that function homologously to prokaryotic VKOR proteins [3,21,22,23] (See also Bevans *et al.* [20] in this Special Issue). Thus, VKOR homologs appear to have evolved very early in vertebrate evolutionary history and apparently carry out critical functions for most species, given their ubiquity and high degree of evolutionary conservation.

#### 1.1.1. VKOR Enzymes Can Catalyze Multiple Reactions

Although the VKOR family is named for the first confirmed function of the human, rat and mouse orthologs [2,24], biochemical characterizations of non-vertebrate homologs reported to-date have indicated that they cannot catalyze VKOR activity, but alternatively catalyze vitamin K quinone reductase (VKR) or ubiquinone reductase activities [25,26,27,28]. To date, only one prokaryotic VKOR homolog from *Mycobacterium tuberculosis* has been demonstrated to possess VKOR activity *in vitro* when expressed in HEK 293 cells [27]. However, the native *M. tuberculosis* lipidome has been shown to possess only quinone and hydroquinone forms of menaquinones, but not menaquinone 2,3-epoxides [29], so it is not likely that the VKOR homolog of this bacterium catalyzes physiological VKOR activity *in vivo*. Subsequent to the initial reports identifying human VKORC1 by virtue of its VKOR de-epoxidase activity, the same enzyme was shown to additionally catalyze *in vitro* VKR activity that reduces vitamin K quinone (K) to vitamin K hydroquinone (KH_2_) [30]. More recently, human VKORC1L1 was also confirmed to catalyze VKOR and VKR activities *in vitro* [31]. Thus, both vertebrate VKOR paralogs catalyze both VKOR and VKR enzymatic activities.

Four cysteine residues (human VKORC1 sequence numbering: Cys43, Cys51, Cys132, Cys135) are completely conserved among VKORC1 orthologs and are required for *in vivo* VKOR catalysis [27,32,33]. Only one *in vitro* study has investigated VKR enzymatic activity for VKORC1 and confirmed that Cys132 and Cys135 are required [30]. Additionally, a conserved serine or threonine (human VKORC1 sequence numbering: Ser57) has been shown to be essential for VKOR catalytic activity *in vitro* [32,34]. Based on sequence homology to the bacterial VKOR enzyme structure, the four redox-active cysteines are widely believed to be arranged in a double disulfide relay that shuttles reducing equivalents from ER-resident oxidoreductases, responsible for *de novo* oxidative protein folding (OPF), to membrane-soluble K>O [30,33,34,35,36]. Thus, VKORC1 accepts reducing equivalents from cysteine thiol groups of soluble oxidoreductase proteins in the ER lumen. Protein disulfide isomerase (PDI), TMX, TMX4 and ERp18, all with thioredoxin-like protein folds, have been implicated as physiological oxidoreductase partners by their ability to form intermolecular disulfide bonds with VKORC1 in cell culture experiments [36,37]. These ER-resident accessory oxidoreductases serve as the primary enzymes that interact with proteins and peptides undergoing oxidative folding by *de novo* disulfide formation [38]. Additionally, VKOR enzymes are the only OPF oxidoreductases that do not ultimately require molecular oxygen as the terminal electron acceptor by downstream enzymatic pathways, suggesting that the origin of these eukaryotic proteins may be very ancient, possibly having evolved before earth’s atmosphere was substantially aerobic, and might predate the evolution of other enzymes involved in OPF in the ER including members of the Ero1, peroxiredoxin, and QSOX families [31,39].

#### 1.1.2. Known Biological Roles for VKOR Family Enzymes

The first biological function attributed to VKORC1 was VKOR catalysis—the rate-limiting step in the classical vitamin K cycle [2,24,40,41]. In humans and other vertebrates, the vitamin K cycle drives post-translational modification of glutamic acid residues to form γ-carboxyglutamyl residues required for proper function of VKD proteins [42,43]. VKORC1 is the sole enzyme in vertebrates capable of sustaining sufficient VKOR activity to maintain hemostasis [42,44]. VKORC1L1 is apparently responsible for other functions as it cannot rescue VKORC1-specific production of VKD clotting factors in *vkorc1^−/−^* knock-out mice [44]. Newborn *vkorc1^−/−^* mice typically died within several days due to internal hemorrhage due to severe deficiency of γ-glutamyl carboxylated clotting factors. The lethal phenotype could be rescued by administration of large doses of vitamin K, similar to the rescue of the human VKCFD2 phenotype in patients homozygous for a VKORC1:Arg98Trp mutation [45]. Interestingly, with respect to hemostatic phenotype, heterozygous *vkorc1^+^*^/*−*^ mice were indistinguishable from homozygous wild-type mice, suggesting that one wild-type *vkorc1* allele is sufficient for producing adequate levels of γ-glutamyl carboxylated VKD clotting factors to sustain normal development and growth. In contrast, with respect to bone morphology, eight-day old *vkorc1^−^*^/*−*^ mice were found to have a pathological phenotype, whereby long bones were all found to be significantly shorter compared to those of homozygous wild-type *vkorc1* mice. In its fully γ-glutamyl carboxylated form, the VKD protein osteocalcin, secreted by osteoblast cells, has long been implicated in bone calcification and homeostasis [46]. Intriguingly, a recent study by Ferron *et al.* (2015) found that VKORC1L1 could not functionally substitute for VKORC1 in cultured osteoblast cells where VKORC1 expression level correlates with γ-glutamyl carboxylation of osteocalcin and modulation of its endocrine functions [47]. Thus, it appears that osteocalcin mediation of bone formation is a second example where VKORC1L1 cannot substitute for VKORC1-mediated biological function of a secreted VKD protein.

In addition to hemostatic functions of VKD clotting factors, other VKD proteins play crucial roles in bone growth and homeostasis [7,13], and recently were demonstrated to be necessary for inhibition of calcification in vasculature [9,10,12,48,49]. Vitamin K and VKD proteins have also been shown to protect oligodendrocytes and neurons from oxidative injury [50], function in cell signaling and growth [15,51], and support sphingomyelin synthesis and metabolism in nervous tissues [14].

A second important biological function was recently confirmed for VKORC1 as an acceptor of reducing equivalents from cysteines during oxidative protein folding in the ER [39]. This was independently confirmed by both siRNA silencing and warfarin knock-down of VKOR enzymatic activity in human hepatoma HepG2 cells after Ero1 α/β isoforms and peroxiredoxin IV (PRDX4) were first functionally silenced, demonstrating that VKORC1 alone can facilitate OPF.

That both vertebrate VKOR paralogs catalyze both VKOR and VKR reactions suggests that neofunctionalization of one of the evolved paralog enzymes, relative to the other retaining an ancestral function, has not occurred—at least with respect to catalytic reactions and substrates. Thus, further elucidation of biological functions for both enzymes may give clues to heretofore-unknown functional differences that might be the basis for selective pressure to conserve their otherwise redundant enzymatic activities in vertebrates. For example, there might be paralog-specific differences in which partner oxidoreductases pass reducing equivalents to each paralog or differences in tissue-specific or developmental stage-specific expression.

As a corollary to the above examples where VKORC1L1 cannot substitute for some of the biological functions of VKORC1 *in vivo*, we ask the question: Are there biological functions mediated by VKORC1L1 that VKORC1 cannot fulfill? Unfortunately, this question has not yet been experimentally addressed as it necessarily requires knock-down or knock-out of VKORC1L1 in cells or animal models that can be used to investigate its biological function. Recently, however, two lines of investigation have begun to focus on details of VKORC1L1 function.

First, a study by Westhofen *et al.* (2011) provided evidence from a number of different experimental perspectives. Expression of VKORC1L1 in HEK 293 T cells in the presence of vitamin K was found to promote vitamin K-dependent cell viability, elimination of intracellular reactive oxygen species and prevented oxidative damage to membrane proteins [31].

Second, a recent comprehensive study by Hammed *et al.* (2013) further measured and compared differential expression of vkorc1 and vkorc1l1 paralogs and tissue-specific VKOR activity of both paralogs in wild-type mice and the *vkorc1*^−/−^ mouse line originally reported by Spohn *et al.* (2009) [44,52]. Expression levels of vkorc1l1 in all tissues investigated were not different in *vkorc1^−^*^/*−*^ mice compared to mice with homozygous wild-type *vkorc1*. Thus, it appears that regulation of vkorc1l1 expression in mice is not sensitive to the level of vkorc1 expression, suggesting that the regulation of expression for both paralogs involves independent regulatory pathways. Furthermore, *in vitro* investigation of VKOR enzymatics for mouse and human VKOR paralog enzymes heterologously expressed in *Pichia pastoris* yielded surprising and unexpected results. While the Michaelis–Menton constants for K_1_ > O were determined to be similar for human VKORC1L1 and VKORC1 and for rat vkorc1l1 and vkorc1 (Table 1), the warfarin inhibition constants (K_i_) for human VKORC1L1 and rat vkorc1l1 were found to be, respectively, 29-fold and 54-fold greater than for the respective VKORC1 and vkorc1 paralogs. Thus, it appears that both human and rat VKORC1L1 paralogs are ~1.5 orders of magnitude less warfarin sensitive than the respective VKORC1 paralogs. Based on these results, the study went on to show that tissue-specific expression of both paralogs contributes to overall level of VKOR activity (*i.e.*, tissue-specific VKOR activities of both paralogs are additive) and that the degree of warfarin sensitivity in various tissues is a function of the relative paralog expression ratio. Interestingly, by use of c-myc tagged expression constructs in *Pichia pastoris* cells, the authors were able to determine that the relative VKOR catalytic efficiency of rat vkorc1 is 30-fold greater than for rat vkorc1l1, while the VKOR catalytic efficiency of human VKORC1 is two-fold lower than that of human VKORC1L1. In summary, the study by Hammed *et al.* has demonstrated that VKORC1L1 is able to support VKOR activity and may constitute an alternative pathway that is able to substitute or partially complement for loss of VKORC1 function in various non-hepatic tissues of *vkorc1^−^*^/*−*^ mice.

#### 1.1.3. Evolutionary Origins of the VKORC1 and VKORC1L1 Paralogs

**Figure 2:**
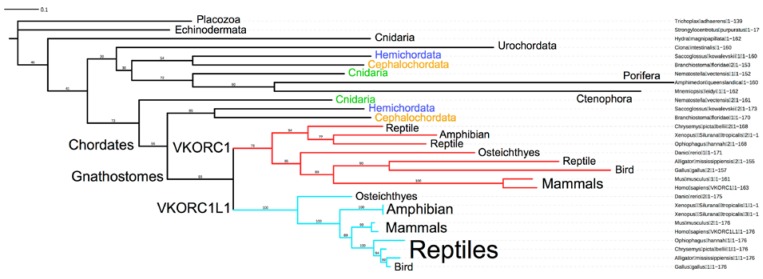
Reconstructed phylogeny (unrooted) for full-length sequences of index metazoans using PhyML with a WAG+I+G4 substitution model in the IQ-TREE ver1.2.3 server-based phylogenetics package with graphics generated using the iToL server (see Bevans *et al.* [20] in this Special Issue for details). Linnaean taxonomic names and primary sequence lengths are shown to the right; groupings shown on the left: Placozoa, basal invertebrate outgroup; Echinodermata and Hemichordata, basal deuterostomes belonging to the Ambulacaria; Cnidaria, basal metazoan invertebrates including jellyfish and sea anemonae; Urochordata, an invertebrate sister group to vertebrates; Porifera, basal non-metazoan animals including sponges; Ctenophora, basal non-bileterian metazoans including comb jellies; Cephalochordata, a basal chordate sister group to Olfactores which includes Urochordata and vertebrates; Chordates includes Cephalochordates, Tunicates (here represented by the Urochordate Ciona intestinalis) and Vertebrates; Gnathostomes are a subgroup of vertebrates with jaw bones that includes Chondrichthyes (sharks and rays—not included among the analyzed sequences; see Note 2 [54]) and Osteichthyes (bony fishes and tetrapods); VKORC1 and VKORC1L1 are paralog clades for Gnathostomes. Scale bar (upper left, labeled 0.1) represents single nucleotide substitution rate per million years; VKORC1 clade is represented by red lines, VKORC1L1 clade by cyan lines; numbers on branches are % support for 1000 bootstrap trees. VKOR paralogs encoded by non-gnathostome genomes are shown in blue (Hemichordata), orange (Cephalochordata) and green (Cnidaria) (see also Note 3 [55]).

Robertson (2004) previously suggested that an ancestral VKOR gene duplication likely occurred in early vertebrates and resulted in the extant human and other gnathostome VKOR paralogs [3]. This would be in agreement with the divergence of the common ancestor of the jawed vertebrates (gnathostomes) from urochordates and cephalochordates, as has been suggested for many other vertebrate protein paralog pairs [53]. In the article by Bevans *et al.* [20] in this special issue, a broad phylogenetic study of VKOR family homologs yielded strong support for distinct monophyletic clades comprising vertebrate VKORC1 and VKORC1L1 homologs. Thus, it is likely that the paralogs arose one time and quickly became fixed in the genomes of subsequently diverged early (crown) vertebrate lineages. That all extant gnathostome genomes sequenced to date include both paralog genes suggests that the functions of both paralogs are indispensable to vertebrate life. In order to more accurately confirm the divergence point of the last common ancestor of modern vertebrates with two VKOR family paralogs, we chose a series of index genomes sampling various evolutionary groupings that diverged before, during and after the last universal common ancestor of gnathostomes, many only very recently sequenced in draft form, to reconstruct a likely metazoan VKOR phylogeny (Figure 2).

As expected, we found high phylogenetic support for monophyletic VKORC1 and VKORC1L1 clades, which split uniformly for extant ganthostomes. Branch lengths for the VKORC1L1 clade (cyan) are relatively shorter than those for the VKORC1 clade (red), in agreement with earlier reports that primary sequences among VKORC1L1 orthologs are more highly conserved than for those among VKORC1 orthologs [2,3]. Reptilian VKORC1L1 orthologs appear on mixed branches with VKORC1L1 orthologs of amphibian, fish and bird as branch support for the VKORC1L1 clade is significantly weaker than for the VKORC1 clade. Surprisingly, for single genomes representing three non-gnathostome groups (Figure 2, indicated in blue, orange and green), we found pairs of VKOR paralogs where one paralog in each genome is inferred to be more similar to the gnathostome VKORs and the second paralogs for each genome are clustered together on a deeper-lying branch. However, inference support for the lower-lying branches (Figure 2, black lines) is considerably lower than the relatively high support for the gnathostome paralog clades. This is evident in the scrambled placement of representative non-gnathostome species in the tree that does not correlate well with the current consensus groupings on the Tree of Life (e.g., Echinodermata is placed on a low branch parallel to Placozoa, and chordate paralogs (Figure 2, blue and orange) are mixed with invertebrate VKOR sequences on a single, deep branch (includes fourth through ninth sequences from the top). Notable results of our phylogenetic analysis include the VKOR paralog pairs of two invertebrate genomes (acorn worm, *Saccoglossus kowalevskii*; lancelet, *Branchiostoma floridae*) that are placed as basal deuterostomes, far deeper in the Tree of Life than vertebrates. This begs consideration that VKOR gene duplications may have occurred in these ancient invertebrate branches independent of the first whole genome duplication in gnathostomes, which, consistent with our inferred VKOR phylogeny, is the likeliest single event that resulted in the gnathostome paralogs. Similarly, the VKOR paralogs found in the Cnidarian sea anemone (*Nematostella vectensis*) may have arisen by a gene duplication unrelated to the gnathostome event. Whether these invertebrate genomes with VKOR paralog pairs represent isolated exceptions, or are evidence for deeper-rooted single gene duplication/loss events, will require more whole genome data from current and future sequencing efforts. In summary, our phylogenetic results suggest that the extant human VKOR paralogs VKORC1 and VKORC1L1 likely arose in an older common metazoan ancestor than the last universal common ancestor of gnathostomes, likely as early as the common ancestor of crown chordate groups.

## 2. Common Aspects of VKORC1 and VKORC1L1 Structure and Function

### 2.1. Gene and Protein Structural Organization

Parsing vertebrate *VKORC1* and *VKORC1L1* sequences in the NCBI Gene database confirmed both paralogs are organized into three exons of very similar lengths. Intron lengths vary considerably between the two paralogs with entire VKORC1L1 genes being typically 17–25 times longer than the respective VKORC1 paralogs (e.g., VKORC1: 2.3 kb mouse—human 3.5 kb; VKORC1L1: 40 kb mouse—86 kb human). In contrast, Robertson (2004) noted that three kinetoplast VKOR homologs, *Trypanosoma cruzi*, *T. brucei*, and *Leishmania major*, are encoded by single exon genes [3]. Pseudogenes found in the human, mouse and rat genomes have been previously reviewed in detail [3].

**Figure 3:**
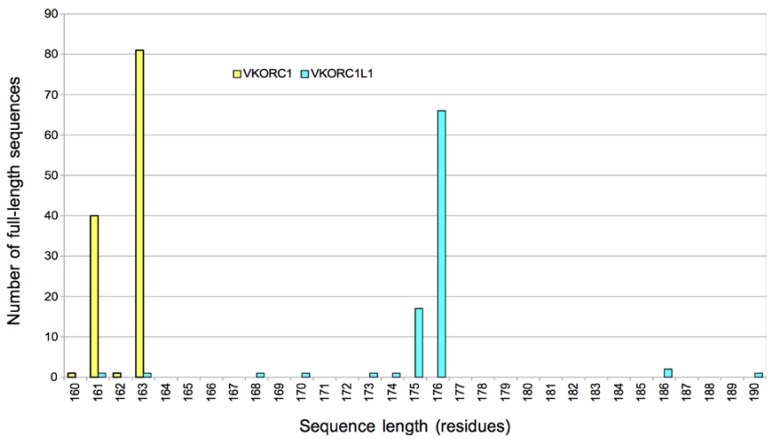
Histogram of vertebrate VKOR paralog sequence lengths in the NCBI Protein database. All sequences included were verified as full-length isoform 1 for VKORC1 (yellow bars) and VKORC1L1 (cyan bars).

Inspection of vertebrate VKORC1 and VKORC1L1 full-length (isoform 1) protein sequences in the NCBI Proteins database revealed that most vertebrate VKORC1 orthologs are about 161–163 residues (Figure 3, yellow bars; range 160–163 residues), whereas VKORC1L1 sequences are predominantly 174–176 residues (Figure 3, cyan bars; range 161–190 residues). Most vertebrate VKORC1 ortholog primary sequences encompass a core domain of 153 residues (Figure 1, human VKORC1 residues Met1-Val153) with a *C*-terminus of variable length (7–14 residues). All VKORC1L1 sequences include an additional 3-residue insertion between corresponding human VKORC1 residues 10 and 11 (Figure 1, human VKORC1L1 residues Arg19-Tyr20-Ala21), effectively extending the length of the predicted 1st TMH by one α-helical turn and resulting in a core domain length of 152 residues (Figure 1, human VKORC1L1 residues Pro12-Leu163). The variable length *N*-termini of VKORC1L1 orthologs are 1–63 residues with the majority of orthologs having an *N*-terminal length of 11 residues. *C*-termini of VKORC1L1 orthologs are 8–13 residues with the majority having a length of 13 residues. Both VKORC1 and VKORC1L1 are localized to and retained in the ER [2,31], likely by a COP I-mediated mechanism of the *cis*-Golgi that recognizes known ER retention recognition sequences with adjacent pairs of positively charged amino acids in the *C*-termini of membrane-intrinsic proteins [56]. Recently, an additional ER retention motif in the short cytoplasmic loop connecting TMH2 and TMH3 of human VKORC1 has been identified [57].

Extramembraneous loops are of identical length among all sequenced vertebrate VKOR homologs. Thus, respective VKORC1 and VKORC1L1 proteins in vertebrates are expected to have highly homologous, evolutionarily conserved respective protein folds.

### 2.2. In Vitro VKOR Enzymatics—Substrates and Inhibitors

Although the first detailed enzymatic studies of VKOR activity in liver microsomes prepared from mice and rats commenced in 1984 [58,59,60], more recent studies, since 2011, characterizing the enzymatics of recombinantly produced human and rat VKORC1 and VKORC1L1 are just now gaining momentum among several active, independent research groups [31,52,61,62,63,64,65,66,67,68]. In order to form a comprehensive picture of our current understanding of VKOR enzymatics, we have summarized the basic results of these studies (Table 1). Of the dozen studies specifically addressing VKOR enzymatics, the initial three relied on rodent liver microsomal preparations as enzyme sources which, during preparation, substantially lose the ER lumenal oxidoreductases that are required for physiological VKOR activity *in vivo* [58]. In order to supply reducing equivalents to drive VKOR activity *in vitro*, DTT has been widely used since it was found to support VKOR activity (for a historical review of the DTT-driven *in vitro* VKOR assay, see [68]). Thus, in the DTT-driven VKOR assay, VKORC1 or VKORC1L1 catalyze reduction of K>O with concomitant oxidation of DTT. To achieve this, the enzymes function by a kinetic mechanism that alternates between two states where the active site CXXC motif cysteines are either oxidized (in the form of a disulfide bridge between them) or reduced [58,63,65,69]. Two different enzyme kinetic models have thus far been applied to VKOR studies—the “ping-pong” model takes enzymatic conversion of both substrates into account [58,63,65,68], while a simpler Michaelis–Menton single substrate kinetic model is based on enzymatic conversion of only the K>O substrate [31,52,59,60,61,62,64,66,67]. For the K>O single substrate model to be valid, the DTT substrate must be at saturating concentration in the VKOR assay a requisite condition for pseudo first-order kinetics [70]. Both kinetic models can yield valid enzymatic parameters (e.g., K_m_, V_max_, k_cat_) from DTT-driven VKOR assay data. However, due to the fact that DTT competes with warfarin for binding to the enzymes, interpretation of warfarin dose-response data obtained using the DTT-driven VKOR assay has been extremely problematic [63,65,71,72]. For example, VKORC1 variants with single amino acid mutations that cause warfarin resistance in humans and rodents show dose-response data indicating warfarin susceptibility identical to wild-type VKORC1 [2,73]. This problem in *in vitro* assessment of resistance phenotypes for known VKORC1 warfarin-resistant variants has been recently overcome by use of alternative cell culture-based VKOR activity assays (see below) which yield warfarin resistance dose-response data in agreement with human and rodent resistance phenotypes. However, an advantage in continuing use of non-physiological reductant-driven VKOR assays lies in their ability to provide data suitable for detailed enzymatics and catalysis mechanism studies since, unlike in cell culture-based assays, the assay conditions can be strictly defined.

What we can generally conclude from VKOR enzymatics studies to-date includes (referring to Table 1): (1) wild-type VKORC1 and VKORC1L1 Michaelis-Menton constant (K_m_) values determined for K>O substrates are in the low micromolar (~1–35 μM) range, while K_m_ for DTT and THPP reducing substrates are approximately millimolar (V_max_ values are not comparable between studies as they reflect a convolution of intrinsic enzyme turnover rate with the quantitative amount of enzyme used in the assay); (2) the enzymes to not appear to significantly discriminate between phylloquinone- and menaquinone-2,3-epoxide substrates; (3) for all warfarin resistance mutations studied, except for Tyr139 position mutations in rats, measured K_m_ values for K>O are considerably greater than for the respective wild-type enzymes, implying K>O substrate binding affinity is diminished by nearly all mutations; (4) the DTT-driven VKOR assay reveals warfarin-resistant *in vitro* phenotypes only for a very few mutations investigated (Table 1; last column, K_i_ values indicated in bold-face type are significantly increased with respect to the wild-type K_i_ in each study); and (5) both human and rat VKORC1L1 enzymes appear to be considerably less warfarin-sensitive than the respective VKORC1 enzymes. To-date, enzymatic studies of VKR catalytic function for VKORC1 and VKORC1L1 have not been reported. From enzymatic study of VKOR catalysis available so far for both VKORC1 and VKORC1L1, we are nudged towards the conclusion that there is no major difference in enzymatic function or substrate specificity between the vertebrate VKORC1 and VKORC1L1 paralogs.

**Table 1:** Summary of results from published VKORC1 and VKORC1L1 enzymatics studies.

Study	Enzyme	Species	K_m_ (K_1_ > O) (μM)	K_m_ (K_2_ > O) (μM)	K_m_ (DTT) (mM)	pH	K_m_ (THPP_Total_) (μM)	K_i_ (warfarin) (μM)
Krettler *et al.* 2015 [65]	r VKORC1	human	**1.20**			7.4	431	
Goulois *et al.* 2015 [64]	r vkorc1	*R. rattus*	**15.9 ± 4.5**			7.4		0.32 ± 0.07
	r vkorc1	*R. norvegicus*				7.4		0.50 ± 0.01
	r vkorc1:Y25F	*R. rattus*	**15.9 ± 4.5**			7.4		**1.99**
Matagrin *et al.* 2014 [63]	r VKORC1	human				7.4		1.65
Hammed *et al.* 2013 [51]	r VKORC1	human	**21.5 ± 4.2**			7.4		1.8 ± 0.2
	r vkorc1	rat	**19.6 ± 1.6**			7.4		0.6 ± 0.04
	r VKORC1L1	human	**24.1 ± 3.0**			7.4		52.0 ± 3.0
	r vkorc1l11	rat	**35.0 ± 3.0**			7.4		32.6 ± 1.9
Matagrin *et al.* 2013 [61]	r vkorc1	rat	**7.2 ± 2.5**			7.4		
	r vkorc1:L120Q	rat	25.0 ± 4.0			7.4		
	r vkorc1:L128Q	rat	12.1 ± 1.0			7.4		
	r vkorc1:Y139C	rat	60.0 ± 6.0			7.4		
	r vkorc1:Y139F	rat	17.8 ± 4.5			7.4		
	r vkorc1:Y139S	rat	13.1 ± 1.3			7.4		
Bevans *et al.* 2013 [60]	r VKORC1	human	1.24		8.38	7.5		2.481
						7.5		2.633
						7.5		5.786
Hodroge *et al.* 2012 [59]	r VKORC1	human	**19.8 ± 4.5**			7.4		1.65 ± 0.79
	r VKORC1:A26P	human	57.4 ± 10.1			7.4		**18.43 ± 5.82**
	r VKORC1:A26T	human	18.7 ± 1.4			7.4		2.13 ± 0.56
	r VKORC1:L27V	human	22.8 ± 2.9			7.4		1.83 ± 0.62
	r VKORC1:H28Q	human	29.8 ± 4.6			7.4		0.65 ± 0.42
	r VKORC1:D36G	human	43.8 ± 0.2			7.4		0.74 ± 0.25
	r VKORC1:D36Y	human	23.6 ± 0.2			7.4		1.82 ± 0.70
	r VKORC1:A41S	human	65.9 ± 5.4			7.4		1.78 ± 0.02
	r VKORC1:V45A	human	26.9 ± 2.3			7.4		1.10 ± 0.04
	r VKORC1V54L	human	102.5 ± 28.6			7.4		**7.95 ± 1.32**
	r VKORC1:S56F	human	23.2 ± 6.2			7.4		1.05 ± 0.82
	r VKORC1:R58G	human	71.0 ± 10.9			7.4		1.50 ± 0.36
	r VKORC1:W59C	human	179.7 ± 12.5			7.4		1.16 ± 0.20
	r VKORC1:H68Y	human	16.9 ± 2.8			7.4		**6.21 ± 0.85**
	r VKORC1:I123N	human	27.0 ± 2.1			7.4		**4.01 ± 1.01**
	r VKORC1:Y139H	human	9.2 ± 3.0			7.4		**5.91 ± 1.77**
Hodroge *et al.* 2011 [58]	r vkorc1	rat	**7.20 ± 2.50**			7.4		0.50 ± 0.05
	vkorc1^wt/wt^	rat	**8.40 ± 0.90**			7.4		0.72 ± 0.01
	r vkorc1:L120Q	rat				7.4		**>100**
	r vkorc1:L128Q	rat				7.4		**4.0 ± 0.7**
	r vkorc1:Y139C	rat				7.4		**>100**
	r vkorc1:Y139F	rat	17.8 ± 4.5			7.4		**>100**
	vkorc1:Y139F^+/+^	rat	19.5 ± 4.0			7.4		**29.0 ± 4.1**
	r vkorc1:Y139S	rat				7.4		**>100**
Westhofen *et al.* 2011 [30]	r VKORC1	human	**1.88 ± 0.13**	**1.55 ± 0.55**		7.6		
	r VKORC1L1	human	**4.15 ± 0.10**	**11.24 ± 0.23**		7.6		
Lasseur *et al.* 2006 [57]	vkorc1^wt/wt^	mouse	**12.73 ± 0.93**			7.4		5.97 ± 0.38
	vkorc1^W59G/W59G^	mouse	15.31 ± 4.92			7.4		3.5 ± 0.27
Lasseur *et al.* 2005 [56]	vkorc1^wt/wt^	rat	**57.7 ± 12.5**			7.4		0.72 ± 0.06
	vkorc1^Y139F/Y139F^	rat	19.5 ± 4			7.4		**29 ± 4.1**
Hildebrandt *et al.* 1984 [55]	vkorc1^wt/wt^	rat	10.0 ± 0.7 *		0.60 ± 0.03	8.8		
	vkorc1^wt/wt^	rat	9.1 ± 0.13 *		0.54 ± 0.04	8.8		
	vkorc1:^Y139S/Y139S^	rat	4 *		0.16	8.8		
	vkorc1^wt/wt^	rat	9		0.43	7.2		
	vkorc1:^Y139S/Y139S^	rat	6		0.29	7.2		

Symbols: r, (Enzyme column) recombinantly produced enzyme; * (K_m_ (K_1_ > O) column), sodium cholate-solubilized and partially purified enzyme. All enzymes are wild-type unless indicated by a colon followed by a specific mutation; samples prepared from liver microsomes are indicated with a superscript where individual *VKORC1* alleles (separated by a slash) are indicated as (wild-type) or a specific mutation. Bold-face type in columns for Michaelis–Menton constants (K_m_ values) indicates values for wild-type enzymes. Bold-face type in the column for warfarin inhibition constants (K_i_) indicates warfarin resistance phenotypes confirmed by *in vitro* measurement.

### 2.3. In Vitro Cell Culture-Based Assays of VKOR Activity

Recent studies have confirmed *in vitro* warfarin resistance phenotypes for known human VKORC1 mutations that are in agreement with reported *in vivo* resistance phenotypes [74]. Compared to warfarin IC_50_ values for wild-type warfarin-sensitive and warfarin-resistant human VKORC1 variants determined by Fregin *et al.* (2013) [75] and Czogalla *et al.* (2013) [76], warfarin IC_50_ values determined in the study of Tie *et al.* (2013) are all about an order of magnitude lower, but all of these studies ranked *in vitro* phenotype severity, with respect to specific mutations, identically [77]. Although the purpose of the study by Haque *et al.* (2014) was to investigate dose-response for warfarin and its hydroxylated metabolites, and did not investigate warfarin-resistant VKORC1 variants, the warfarin IC_50_ value obtained for wild-type human VKORC1 is greater than the values obtained in all of the other studies [78]. Directly compared, warfarin IC_50_ values reported by Fregin *et al.* (2013) and Haque *et al.* (2014) are 6.9-fold and 18.3- to 35.4-fold greater than the value reported by Tie *et al.* (2013) [75,77,78]. Variations in assay conditions between the studies that could account for the differences in warfarin dose-response have not yet been identified. One important difference between the non-physiological reductant-driven VKOR assays and the more physiological cell culture-based assays is that the former directly assess enzyme function, while the latter are actually indirect assays of enzyme function in that they each rely on the rate-limiting function of VKOR enzymes in the vitamin K cycle to ultimately enable intracellular γ-gutamyl carboxylation of VKD proteins heterologously coexpressed by the cells and secreted into the culture medium. Thus, while the effects of warfarin inhibition on the read-out VKD protein status can be directly attributed to warfarin's localized interaction with VKORC1 or VKORC1L1, there are likely many other influences on the secreted VKD status (e.g., due to choices of cell line, specific VKD reporter protein, expression vector, and variability in culture medium constituents especially in amounts of warfarin-binding serum albumin, *etc.*) that could have profound influence on the correspondence between applied warfarin dosage and secreted VKD protein response. Balancing these possible uncertainties are the opportunities to use these cell culture-based assays to explore the nature of the enzymes’ biological functions. Thus, identification of native partner oxidoreductases that provide the physiological reducing equivalents to drive VKOR activity and characterizing their interactions with VKORC1 and VKORC1L1 in the ER lumen could be experimentally addressed. Similarly, studies could be designed to elucidate regulation of the respective gene transcription and protein expression for VKOR paralogs in cell lines representative of various native tissues and developmental stages. Cultured cell assays would also be useful in identification and assessment of new pharmacological lead compounds based on vitamin K or intended for use as oral anticoagulants with desired qualities superior to currently available warfarin and other 4-hydroxycoumarin derivatives.

## 3. Differences between VKORC1 and VKORC1L1 Paralogs

### 3.1. Tissue- and Developmental Stage-Specific Expression

It has been known for a long time that liver is the primary location of VKOR enzymatic activity essential to the vitamin K cycle and production and secretion of VKD coagulation factors [35,71]. In 2000, before the identification of the *VKORC1* and *VKORC1L1* genes, a study by Itoh and Onishi investigated developmental changes in VKOR enzymatic activity of human liver sampled from autopsied samples representing individuals from 12 weeks post-fertilization to 18 years of age [79]. They found hepatic VKOR activity was low (mean 100 nmol/15 min/g_liver_) and possibly declined through prenatal week 30, then increased abruptly by prenatal week 35 (mean 200 nmol/15 min/g_liver_) and thereafter remained constant through age 18. With the identification of both VKOR paralogs in 2004, recent discoveries of biological roles for non-coagulation factor VKD proteins, and elucidation of what cells and tissues are their primary sites of expression, an increasing number of studies have been focused on assessing tissue-specific expression distributions for both VKORC1 and VKORC1L1 (Table 2). With respect to developmental expression of vkorc1 in mouse, two studies provided some early insight. Ko *et al.* (1998) constructed a cDNA library from total mRNA prepared from 7.5-day post-conception mouse embryonic and extraembryonic cells and found no evidence of vkorc1 expression by RT-PCR analysis [80]. A subsequent mouse tissue expression study by Diez-Roux *et al.* (2011) used *in situ* RNA hybridization on whole embryo sections and found diffuse, weak expression of vkorc1 by 14.5 day post-fertilization embryos [81].

Two recent studies determined mouse VKOR paralog expression profiles for mouse tissues by relative mRNA expression quantitation using qRT-PCR of cDNA prepared from tissue-specific mRNAs. Hammed *et al.* (2013) measured vkorc1l1 expression in liver, lung, and testis of both C57BL/6 wild-type and *vkorc1^−^*^/*−*^ mice and in wild-type nine week-old OFA-Sprague Dawley rat brain, kidney, liver, lung and testis and rat osteosarcoma cell line ROS17/2.8 (Table 2) [52]. In mouse tissues, vkorc1l1 expression levels were highly similar between wild-type and *vkorc1^−^*^/*−*^ strains, suggesting distinctly independent regulation of expression for both VKOR paralogs. For all tissues investigated, vkorc1 expression was also found, but most predominantly in liver (10-fold greater than for vkorc1l1), whereas brain had greater vkorc1l1 expression relative to that for vkorc1. Other tissues had intermediate expression levels for both paralogs. For expression levels in rat liver, lung, brain, kidney and testis assessed at three, six and nine weeks post-partum, both paralogs showed minor variations that were not statistically significant except for vkorc1 expression in liver which peaked significantly at six weeks before declining at nine weeks. Taken together, these results explain why some extrahepatic tissues may have near physiological VKOR activities and down-stream VKD protein function in the presence of warfarin concentrations that effectively inhibit VKD clotting factor production in the liver. Another study by Caspers *et al.* (2015) similarly investigated VKOR paralog expression levels in 29 different tissues of CD1 wild-type mouse [82]. Expression levels for vkorc1 were found to be greatest in liver, lung and exocrine tissues including mammary, salivary and prostate glands, whereas vkorc1l1 expression was greatest in brain (Table 2). Taken together, results of both studies investigating rodent tissue expression patterns for both VKOR paralogs strongly suggests an emerging picture of independent regulation of the vitamin K cycle by differential expression of both VKOR paralog enzymes.

In zebrafish, Fernández *et al.* (2015) have assessed Vkorc1 and Vkorc1l1 expression by qPCR analysis during larval development and in adult tissues [83]. Vkorc1l1 was expressed at highest levels overall at the post-fertilization 4-cell stage and diminished by Prim-5 stage, remaining stable at later stages. Vkorc1 expression was detectable at the 4-cell stage, but peaked at 72–96 h post-fertilization followed by lower, stable levels at later stages. In adults, Vkorc1l1 was ubiquitously expressed in all tissues investigated (Table 2) with greatest levels in brain, muscle and ovary, while Vkorc1 was only detectable in about half of the surveyed tissues with elevated levels in brain, muscle and vertebra. Interestingly, no Vkorc1 expression could be detected in adult intestine, kidney, ovary, spleen and stomach. Using the ZFB1 cell line developed in a previous study [84], the authors found Vkorc1 and Vkorc1l1 to be significantly overexpressed during differentiation, but not during induction of extracellular matrix (ECM) mineralization, in cells cultured for 1 week. In cells cultured for three weeks, there was no significant difference between differentiating cells or cells induced to mineralize ECM. Taken together with results reported for ROS 17/2.8 osteoblast-like cells by Hammed *et al.* (2013), and moderate expression levels of both VKOR paralogs in vertebral tissue, the authors suggest that osteoblast differentiation may require increased vitamin K cycle turnover [51].

Since the initial draft of the human, mouse and rat genomes completed in 2000, 2002 and 2004, respectively, whole genome and proteome investigations have enabled large-scale, high through-put investigation of gene and protein expression levels in various tissues and cells [85]. Less than a year after the identification of both VKOR paralogs in 2004, high-density nucleotide arrays including *VKORC1* and *VKORC1L1* sequences were already being used to explore gene expression on a genomics scale. CHiP-Seq mRNA quantification studies including data for *VKORC1* and *VKORC1L1* have been published for frog, fruitfly, human, rat, mouse, pig, and zebrafish [86,87,88,89,90].

We recently recovered human and mouse VKOR paralog mRNA expression profiles based on chromatin immunoprecipitation (ChIP-seq) technology from a large, high through-put transcriptomics study by Su *et al.* (2004) available through the BioGPS database portal [86,91]. Their data includes VKORC1 and VKORC1L1 expression levels for 79 human and 61 mouse tissues from pooled samples of typically 1–10 individuals. For simplification, in Table 2 we summarize only results including tissues with the 10 highest VKORC1 and VKORC1L1 expression levels above mean values for all tissues (for comprehensive tissue data, see Figure S1, online Supplemental Material). For human and mouse, VKORC1L1 is uniformly expressed at or near median value for most all tissues and cells surveyed. From among those surveyed, only adipocytes, CD34+ cell lines (including monocytic lines) and B lymphoblasts exhibit statistically significant higher levels of VKORC1L1 expression than the median. Westhofen *et al.* (2011) previously pointed out that all three tissues/cell types generate intensely and protractedly elevated levels of ROS under physiological conditions, suggesting a role for VKORC1L1 in redox homeostasis [31]. BioGPS tissue-specific expression levels for *VKORC1* mRNA exhibit more highly varied differences than for *VKORC1L1*. Among tissues with the highest expression levels are liver, where most of the vitamin K-dependent blood-clotting factors are produced, and adipocytes, smooth muscle, thyroid, lung and pineal body.

Data from the GTEx Portal [92], a large-scale, high through-put human genomics project published earlier this year, has recently been mined for a study by Melé *et al.* (2015) on RNA-seq deep-sequenced transcriptomes of 175 individuals that covers 29 solid organ tissues, 11 brain subregions, whole blood and two standard cell lines [93]. We summarize the top six expressing tissues for each VKOR paralog in this study (Figure S2, online Supplemental Material) for comparison with data from other studies. Tissues with highest levels of VKORC1 (ENSG#167397) expression from the GTEx project data included aorta and coronary artery, liver, pituitary and gland, while VKORC1L1 (ENSG#196715) expression was found to be greatest in adipose tissue, mammary gland, lung and tibial nerve (Table 2). Interestingly, the study results indicated that gene activity, in general, differed substantially more across tissues than across individuals and expression patterns for both *VKORC1* and *VKORC1L1* follow this trend. Genes that changed expression (FDR < 0.05) with age across all GTEx study tissues included *VKORC1L1*, but not *VKORC1* [94].

**Table 2:** Summary of results from expression studies of human, mouse and rat VKORC1 and VKORC1L1.

**Study**	Itoh & Onishi 2000 [76] *	Su *et al*. 2004 [83] *	Hammed *et al.* 2013 [51]	Kim *et al.* 2014 [93]	Wilhelm *et al.* 2014 [94]	Caspers *et al.* 2015 [79]	Melé *et al.* 2015 [90] *	Fernández *et al.* 2015 [80] *
**Species**	Human	Human, Mouse	Mouse, Rat	Human	Human	Mouse	Human	Zebrafish
**Method**	VKOR activity assay	CHiP-Seq	mRNA	Mass Spectroscopy	Mass Spectroscopy	mRNA	mRNA	mRNA
**Tissue/cell types**	**Liver (12 weeks post-fertilization to 18 years)**	**Adipocyte**			**Adipocyte**		**Adipose-visceral** **(omentum)**	
							**Adipose-subcutaneous**	
				**Adrenal gland**	Adrenal gland	Adrenal gland		
							**Aorta**	
					Blood platelet			
					Bone	Bone		Operculum
		**Brain (whole)**	**Brain**		Brain	**Brain**		**Brain**
					Breast	Mammary gland	**Breast-mammary**	
						Caecum		
					Cerebral cortex			
		**Colon**		Colon	Colon	Colon		
					Colonic epithelial cell			
							**Coronary artery**	
						Diaphragm		
						Duodenum		
				Esophagus				
						Eye		Eye
**Tissue/cell types**	**Liver (12 weeks post-fertilization to 18 years)**			**Fetal Brain**				
				Fetal Gut				
				Fetal Heart				
				Fetal Liver				
				Fetal Ovary				
				**Fetal Placenta**				
				**Fetal Testis**				
				Frontal cortex				
				Gallbladder	Gall bladder			
								Gills
					Gut			
				Heart	Heart	Heart		
					Helper T-lymphocyte			
				Hematopoietic B cells				
				Hematopoietic CD4+ T cells				
				Hematopoietic CD8+ T cells				
		**CD34+**						
				**Hematopoietic** **Monocytes**	**Monocyte**			
**Tissue/cell types**	**Liver (12 weeks post-fertilization to 18 years)**			Hematopoietic NK cells				
				**Hematopoietic** **Platelets**				
					Ileum epithelial cell			Intestine
			**Kidney**	Kidney	Kidney	Kidney		Kidney
		**Liver**	**Liver**	**Liver**	Liver	**Liver**	**Liver**	Liver
		**Lung**	**Lung**	**Lung**	Lung	**Lung**	**Lung**	
		**721 B-ymphoblasts**				Lymph node		
							**EBV** **transformed** **lymphocytes**	
						Masseter muscle		
		**Mast-cells-IgE**						
		**Mast-cells-IgE+antigen-1 h**						
		**Mast-cells-IgE+antigen-6 h**						
		**Mega-erythrocyte-progenitor**			Milk			
						Muscle		**Muscle**
					Myometrium			
					Natural killer cell			
							**Nerve-tibial**	
						Oesophagus		
**Tissue/cell types**	**Liver (12 weeks post-fertilization to 18 years)**	**Osteoblast-day 14**						
		**Osteoblast-day 21**						
		**Osteoblast-day 5**						
			**Rat osteosarcoma cell line ROS 17/2.8**		Osteosarcoma cell			
				**Ovary**	Ovary	Ovary		**Ovary**
				Pancreas	**Pancreas**	Pancreas		
					**Pancreatic islet**			
		**Pineal body**						
		**Pituitary**					**Pituitary**	
					Placenta			
					Prefrontal cortex			
				Prostate	Prostate gland	**Prostate**		
				Rectum	Rectum			
				Retina	**Retina**			
						**Salivary gland**		
					Skin	Skin		Skin
		**Smooth-muscle**						
						Soft tissue		
				Spinal cord	Spinal cord			
					Stomach	Stomach		Stomach
						Spleen		Spleen
			**Testis**	**Testis**	**Testis**	Testis		
		**Thyroid**						
**Tissue/cell types**	**Liver (12 weeks post-fertilization to 18 years)**					Tongue		
							**Transformed fibroblasts**	
		**Umbilical-cord**						
				Urinary bladder	Urinary bladder			
						Uterus		
								Vertebra
						Vessels		

Symbols: * only cells and tissues shown with significantly greater expression than mean levels for all cells/tissues included in large-scale study; bold type indicates cells/tissues with expression levels significantly above means for each study; yellow, high VKORC1 expression level; blue, high VKORC1L1 expression, green high VKORC1 and VKORC1L1 coexpression.

Whole proteome studies using mass spectroscopic (MS) technologies have recently provided an opportunity to identify and quantitate intracellular pools of translated proteins across tissue types and populations of individuals [95]. Two recent MS proteomics studies include informative protein expression for human VKOR paralogs. Kim *et al.* (2014) systematically examined 30 different human tissues, including seven fetal tissues and six hematopoietic cell types from rapidly acquired postmortem samples from each of three donors [96]. Results for protein levels (~84% coverage of total predicted human proteome) of VKORC1 and VKORC1L1 from this study are available on the Human Proteome Map server [96]. VKORC1 proteolytic peptides (six 6–18 residue peptides representing 31% primary sequence coverage) were detected in six tissues including adrenal gland, monocytes, platelets, lung ovary and testes, while VKORC1L1 peptides (eleven 7–19 residue peptides representing 41% primary sequence coverage) were detected in fetal brain, placenta, testes and in adult lung (Table 2 lists all tissues in which VKOR paralog peptides were detected by MS). A similar MS proteomics study by Wilhelm *et al.* (2014) combined their own data from a similar number of tissues with >10,000 publicly available MS raw data files to generate a database encompassing 60 human tissues, 147 cell lines and 13 body fluids [97]. This study achieved a record 92% coverage of human proteome ORFs. VKORC1 (four 11–30 residue peptides representing 41.1% primary sequence coverage) was detected at higher than median expression levels only in monocytes, pancrease and retina, while VKORC1L1 (four 11–46 residue peptides representing 51.7% primary sequence coverage) was detected in higher than median expression levels only in brain. Compared to the results of Kim *et al.* (2014), this study had a very low detection efficiency for VKOR paralogs likely due to the much longer proteolytic fragments that were initially generated from tissue samples [96,97]. Comparing both MS studies to ChIP-seq method results (see above), it is clear that the MS-based techniques are still in their infancy as the number of tissues with detectable VKOR paralogs is low compared to the results from transcriptome studies. However, looking across all expression study results (Table 2), we find a general concurrence that VKORC1 is most highly expressed in liver, while VKORC1L1 is most highly expressed in brain.

### 3.2. Promoter Regions of VKORC1 and VKORC1L1 Genes

**Figure 4:**
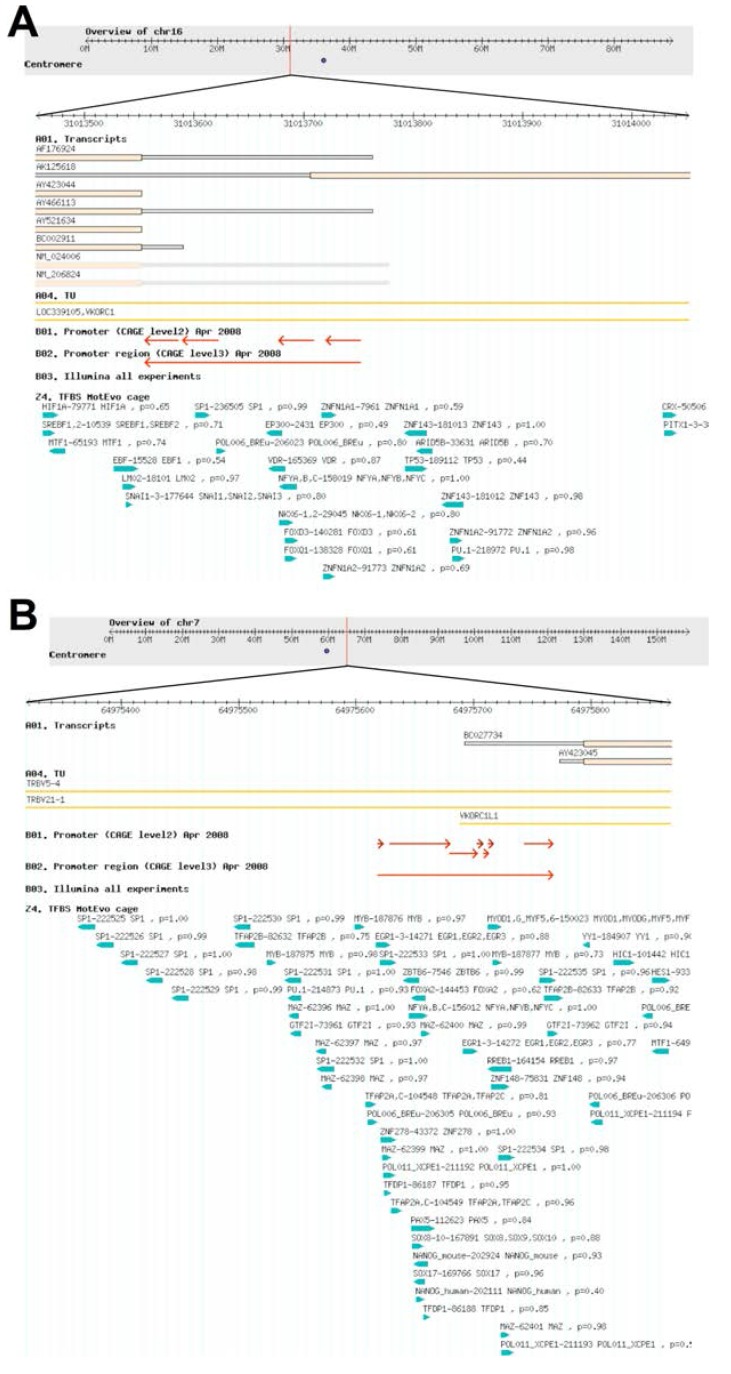
Overview of (top to bottom) chromosome maps (grey fields) showing genetic loci (vertical red lines), alternative mRNA transcripts (A01. Transcripts, tan bars), transcription start sequences (B01. Promoter (CAGE level 2, red arrows) and predicted transcription factor binding sites (24. TFBS MotEvo cage, blue arrows) for (**A**) human *VKORC1*; and (**B**) human *VKORC1L1*. Graphics are from the FANTOM4 Human (hg18) genome viewer (see Note 5 [104]).

Since the coding regions of the *VKORC1* and *VKORC1L1* paralog genes are similarly organized and the respective expressed proteins are so highly conserved that their core domain sequence lengths, predicted folds, catalyzed reactions and substrate usage are essentially identical, we decided to survey existing published data for differences in non-coding regions of the genes for clues to why both paralogs have been preserved with complete fidelity in all extant (*i.e.*, sequenced to-date) vertebrate genomes. In a previously published study of human VKORC1L1 expression and function, we cited tissue-specific ChIP-seq gene expression data for both VKOR paralogs from the Functional ANnoTation Of the Mammalian genome phase 4 (FANTOM4) whole genome expression study [31,98]. For the purpose of exploring similarities and differences of the promoter regions for both human VKOR paralogs, we accessed deep-CAGE data using the FANTOM4 human genome viewer (Figure 4). FANTOM4 focused on the dynamics of transcription start site (TSS) usage in the myeloid cell line THP-1 [99]. We retrieved transcription start site (TSS) and predicted transcription factor binding site (TFBS) data for functionally expressed human *VKORC1* and *VKORC1L1* genes. In summary, *VKORC1* promoter organization is distinctly different from and relatively simpler than that for *VKORC1L1*. *VKORC1* uses four alternative TSSs spanning ~200 bp and 24 predicted TFBSs were identified around this region (Figure 4A, red arrows), while *VKORC1L1* uses seven alternative TSSs spanning ~150 bp with 53 predicted nearby TFBSs (Figure 4B, red arrows).

To predict putative binding sites for known TFBS motifs, a window of −300 to +100 bp flanking each promoter region was extracted, multiply aligned and the MotEvo algorithm applied [100]. Of the 52 predicted transcription regulators, 16 TFs were experimentally confirmed by systematic siRNA knock-down for the *VKORC1L1* promoter region, while only three TFs were found to bind the *VKORC1* promoter region (see Note 4 [101]) [98]. Taken together, these data support the notion that expression of each human VKOR paralog is apparently controlled by distinctly different transcriptional mechanisms, in agreement with previous study results for combined tissue expression levels and respective VKOR enzymatic activities in mice [52,82]. Furthermore, for *VKORC1*, there appear to be many predicted TFBSs in common with other hepatically expressed proteins, while predicted TFBSs for *VKORC1L1* are more similar to those from genes that express proteins with known house-keeping and homeostatic functions [31].

### 3.3. Human Coding Region Mutations

Naturally occurring coding region mutations for human, rat and mouse *VKORC1* genes mostly cause *in vivo* warfarin resistance phenotypes [73,74,102], and one human mutation causes VKCFD2, a severe deficiency in VKD clotting factors [2]. *VKORC1* mutations have been comprehensively reviewed elsewhere [103]. To gain insight into naturally occurring human *VKORC1L1* coding region mutations, we surveyed the NCBI dbVAR database, which includes combined whole genome sequence data for thousands of individuals, and found evidence for 21 total coding region SNPs in living adult humans of which seven are non-synonymous, one premature termination that shortens the *C*-terminus, and six synonymous variants that do not alter translated VKORC1L1 protein sequence. To date, no human *in vivo* phenotypes have been reported for VKORC1L1 non-wild type variants.

### 3.4. Commentary/Hypothesis: Why do Two VKOR Paralogs Persist in Vertebrates?

Here we summarize various conclusions drawn from the studies reviewed in this article and propose a novel hypothesis to explain why two VKOR paralogs with apparently identical enzymatic functions have persisted in vertebrates over ~400 million years of evolution. Rost *et al.* (2004) first noted that VKORC1L1 primary sequences are considerably more conserved among mammalian orthologs than the respective VKORC1 sequences [2]. Robertson (2004) suggested that the extant paralog genes likely arose in a common ancestor before the divergence of urochordates and vertebrates [3]. Phylogenetic analysis we present in this review suggests the last universal common ancestor (LUCA) of all extant metazoans with both VKOR paralogs was likely a crown chordate older than the LUCA of extant gnathostomes (Section 1.1.3. and Figure 2). Given aggregate evidence reviewed in this article that VKORC1 and VKORC1L1 enzymatic functions are virtually identical, but that regulation of developmental stage- and tissue-specific expression is the major notable difference between both paralogs, we further contemplate what other functional differences, at either the protein or biological pathway levels, could provide the required selection pressure to preserve both paralogs.

Addressing this theme, Robertson (2004) commented, “…If this duplication follows the neofunctionalization model of gene duplication (Ohno, 1970) [105], then the rapid divergence of VKORC1 in vertebrates might suggest that its function related to vitamin K recycling might be the derived function, in which case the unknown VKORC1L1 function might better reflect the role of this protein in the other animals and trypanosomatids. Alternatively, if this duplication follows the subfunctionalization model of Lynch & Force (2000) [106], then both proteins might still be involved in vitamin K recycling; however, for some reason VKORC1 has been free to diverge more rapidly in vertebrates than has VKORC1L1…” It is clear from the evidence we review here that neofunctionalization cannot be the driving force for VKOR paralog maintenance, but that subfunctionalization, not of enzymatic function, but of developmental- and tissue-specific expression regulation, might be the basis for this unique preservation of paralogs. Furthermore, we can rationalize a biological need for this beginning with the evolution of aerobic heterotrophic organisms as the earth’s atmosphere became increasingly oxygen-rich through photosynthesis. This led to evolution of multicellular animals and, eventually, vertebrates whose sizes increased over time [19,107]. Accordingly, closed circulatory systems of ever increasing volume and requiring increased cardiac capacity evolved leading to ever increasing circulatory pressure [108]. Parallel to these evolutionary developments in early metazoans, there arose the need for a robust hemostatic system and clotting capability to stem off bleeding through injury [109]. With all of this in mind, we propose that the regulatory mechanisms for VKOR paralog gene expression needed to keep pace with evolutionarily increasing circulatory volume and pressure, and so both VKOR paralogs were maintained in vertebrates due to selection pressure for distinct regulation of expression in non-coding regions of the genes. Thus, VKORC1L1 orthologs may have been maintained for more evolutionarily primitive housekeeping functions that might have included intracellular redox homeostasis and oxidative protein folding under anaerobic growth conditions [31,39]. In contrast, VKORC1 expression regulation has possibly evolved separately to sustain high systemic levels of secreted VKD proteins needed for maintaining large circulatory volume and pressure and also for development and homeostasis of a robust, calcified skeleton.

## 4. Conclusions and Future Perspectives

In this review, we have attempted to comprehensively summarize published results concerning structural and functional similarities and differences for VKORC1 and VKORC1L1 paralogs in extant metazoan genomes and to relate these to a rationale that explains why these proteins are evolutionarily maintained when their enzymatic functions are virtually identical. While it is presently clear that both enzymes are responsible for *de novo* reduction of K vitamins acquired from dietary sources, in addition to recycling oxidized forms of K vitamins to the respective reduced hydroquinone forms in the vitamin K cycle, evolutionary selection pressure has apparently maintained unique physiological functions for both paralogs by a tissue-specific “division of labor” under independent expression and regulatory controls.

In order to address important questions that remain about these paralogs, it will be necessary to more deeply investigate regulation of their expression with respect to cell and tissue type and developmental stage, to identify their functional intracellular protein partners, and to comprehensively identify and characterize new VKD proteins and the extent of the VKD proteome in individual species. We hope this review will stimulate discussion and cooperative investigation among researchers already engaged in vitamin K-related research areas as well as encourage researchers new to the field with expertise in complementary research methods.

## Supplementary Files

Supplementary File 1
