# Supplementary material for: VKORC1 and VKORC1L1: Why do Vertebrates Have Two Vitamin K 2,3-Epoxide Reductases?
Source: Nutrients. 2015 Jul 30;7(8):6250–80. doi: 10.3390/nu7085280 (PMC4555119; doi:10.3390/nu7085280)
Supplement: Supplementary File 1 [file nutrients-07-05280-s001.docx]

Supplementary Information

| 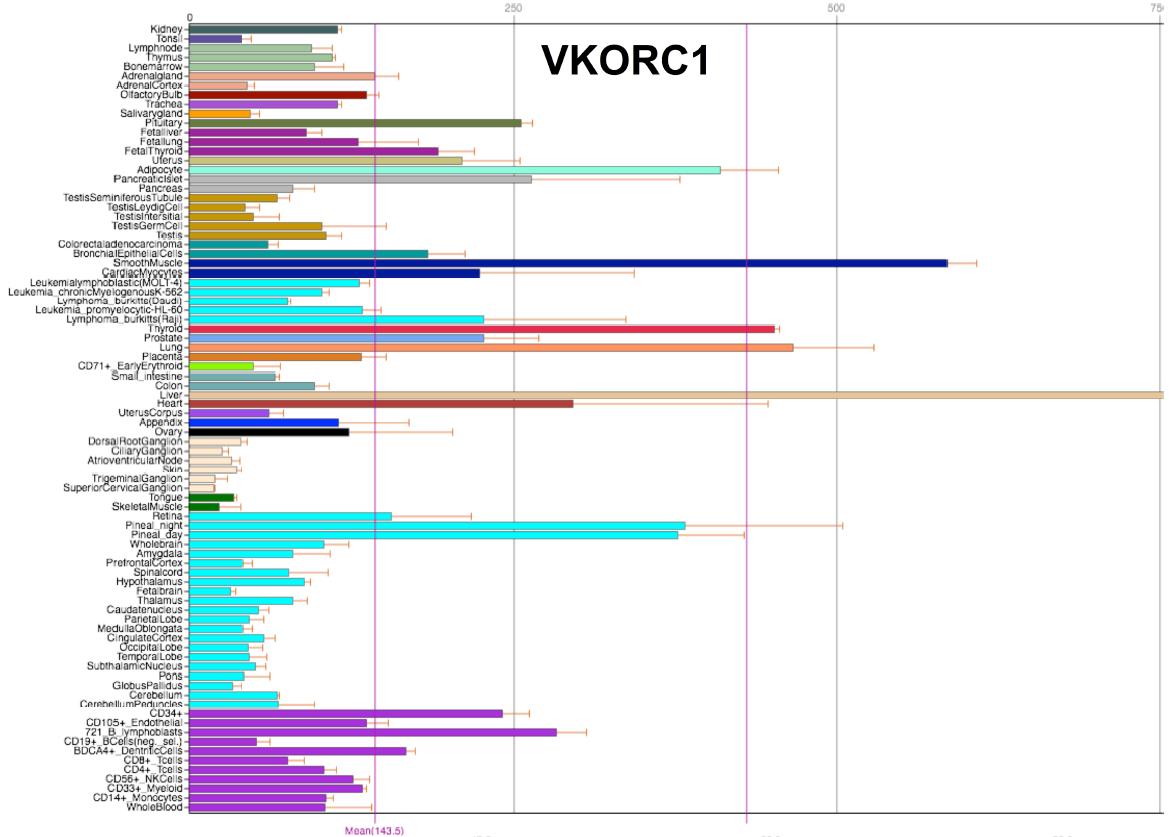 | |
| --- | --- |
| 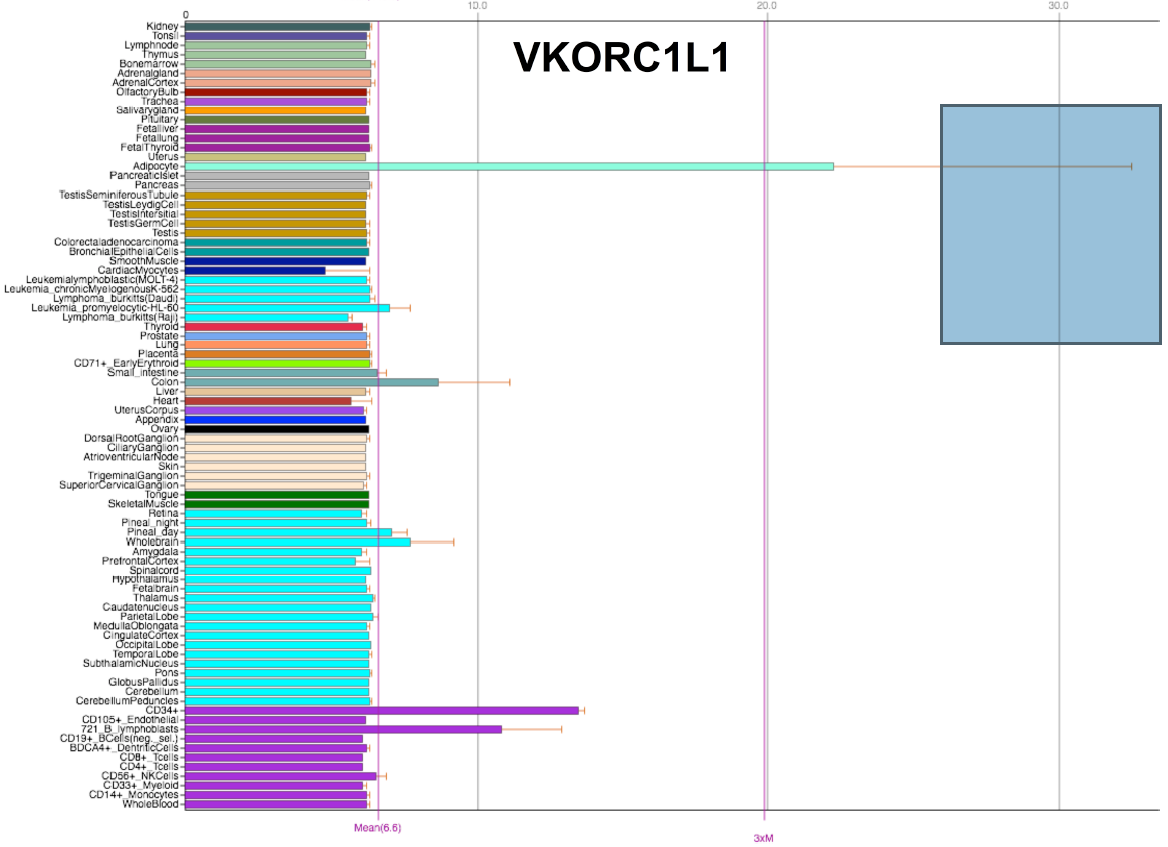 |  |

**Figure S1.** Human tissue-specific expression profiles for VKORC1L1 and VKORC1. (Top) BioGPS tissue-specific expression levels for VKORC1 mRNA exhibit more highly varied differences than for VKORC1L1. Among tissues with the highest expression levels are liver, where most of the vitamin K-dependent blood clotting factors are produced and post-translationally modified by γ-glutamyl carboxylation. (Bottom) Quantitative expression levels for VKORC1L1 mRNA in human tissues and cell populations from the BioGPS gene atlas expression profile database. VKORC1L1 is uniformly expressed at or near median value for most all tissues and cells surveyed. From among those surveyed, only adipocytes, CD34+ cell lines (including monocytic lines) and B lymphoblasts exhibit statistically significant higher levels of VKORC1L1 expression than the median. (http://biogps.gnf.org/, data accession 1 May 2015).


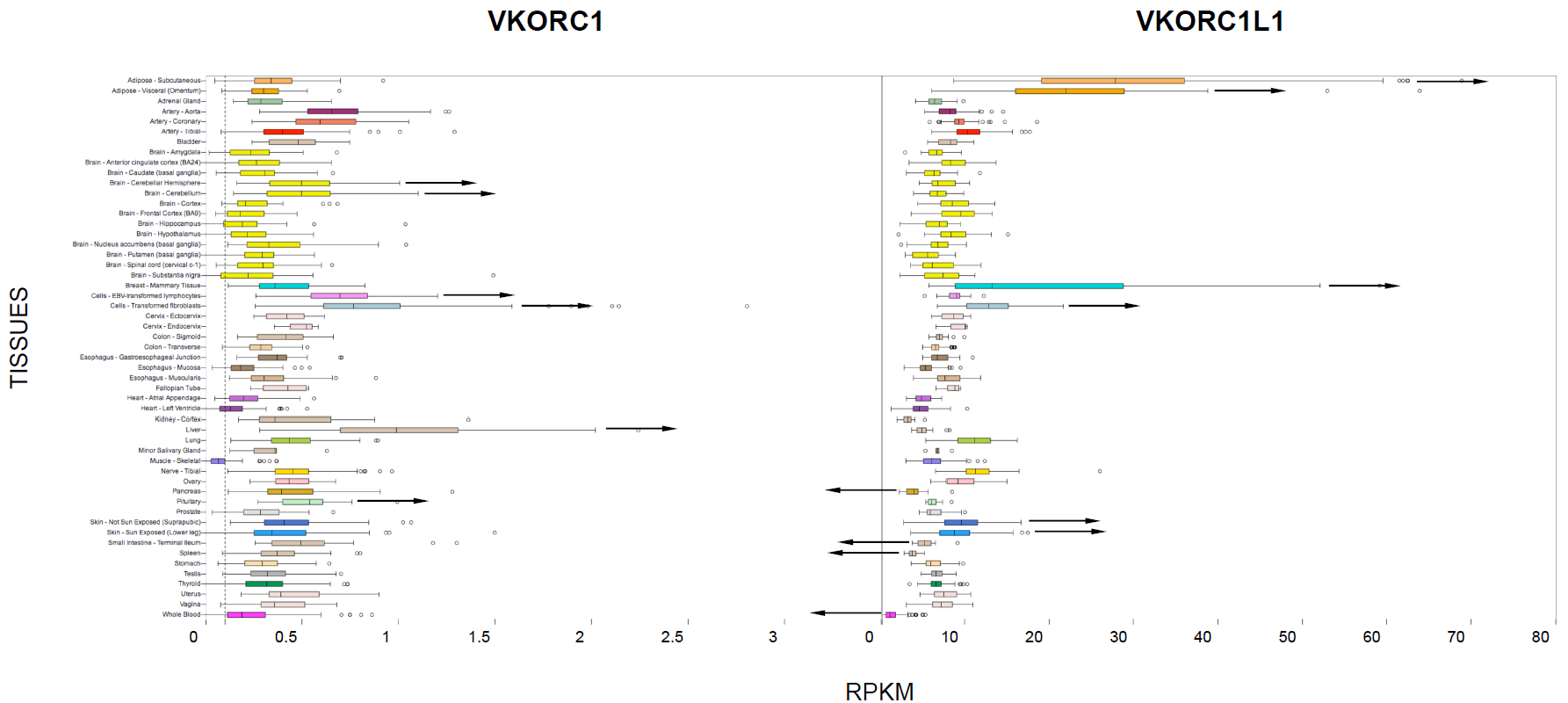


**Figure S2.** Tissue-specific expression profiles from the Genotype-Tissue Expression (GTEx) pilot analysis including (Left) VKORC1 and (Right) VKORC1L1. Expression levels (abscissa) are in reads per kilobase per million mapped reads (RPKM).
